# Intraoperative Measurement of Internal Carotid Artery Blood Flow Using Transit-Time Flowmetry After Carotid Endarterectomy: Results from 196 Consecutive Cases

**DOI:** 10.3390/brainsci16101136

**Published:** 2026-10-21

**Authors:** Meltem Gönel, Corinne Inauen, Philip Heesen, Martina Sebök, Shreyes Paruvacattu, Tristan Schmidlechner, Elisa Colombo, Veronica Percuoco, Jorn Fierstra, Christoph Globas, Susanne Wegener, Andreas Luft, Luca Regli, Giuseppe Esposito

**Affiliations:** 1 Department of Neurosurgery, Clinical Neuroscience Center, University Hospital Zurich, 8091 Zurich, Switzerland; meltem.goenel@usz.ch (M.G.); philip.heesen@uzh.ch (P.H.); martina.seboek@usz.ch (M.S.); shreyes.paruvacattu@uzh.ch (S.P.); tristan.schmidlechner@usz.ch (T.S.); elisa.colombo@usz.ch (E.C.); percuocoveronica@gmail.com (V.P.); jorn.fierstra@usz.ch (J.F.); luca.regli@usz.ch (L.R.); 2 Faculty of Medicine, University of Zurich, 8032 Zurich, Switzerland; susanne.wegener@usz.ch (S.W.); andreas.luft@usz.ch (A.L.); 3 Department of Neurology, Clinical Neuroscience Center, University Hospital Zurich, 8091 Zurich, Switzerland; corinne.inauen@usz.ch (C.I.); christoph.globas@usz.ch (C.G.); 4 Cereneo Center for Neurology and Rehabilitation, 6354 Vitznau, Switzerland

**Keywords:** carotid endarterectomy, transit-time flowmetry, internal carotid artery, intraoperative monitoring

## Abstract

**Highlights:**

**What are the main findings?**

- Transit-time flowmetry is a practical, easy-to-use and quick (<1 min) intraoperative tool for quantitative Internal Carotid Artery (ICA) flow assessment; median intraoperative post-Carotid Endarterectomy (CEA) flow was 170 mL/min (IQR 140–242) in 196 consecutive CEAs.
- Post-CEA ICA flow increases significantly with contralateral steno-occlusive disease severity (*p* < 0.001), reflecting compensatory hyperperfusion.
- Patients requiring intraoperative shunt placement showed higher post-CEA flow (250 vs. 170 mL/min, *p* = 0.010).

**What are the implications of the main findings?**

- TTF provides a quick quantitative intraoperative quality control enabling early detection of inadequate flow restoration.

**Abstract:**

**Background/Objectives**: Carotid endarterectomy (CEA) is a standard surgical treatment for high-grade symptomatic and asymptomatic internal carotid artery (ICA) stenosis. The Transit Time Flowmeter (TTF) provides an easy-to-use tool to quantify intraoperative ICA blood flow in mL/min. This study aimed to (1) describe post-CEA ICA flow quantified with TTF, (2) identify predictors of post-CEA ICA flow, and (3) examine its association with contralateral steno-occlusive disease. **Methods**: We queried data from our prospective registry of patients who underwent a CEA between January 2019 and July 2024 and had intraoperative registered post-CEA ICA flow measurements using TTF. Preoperative ICA stenosis was graded according to the NASCET criteria. The clinical data was retrospectively collected. **Results**: Data from 196 CEAs in 190 patients (median age 74 [67–81]; 25% female) were included. Most patients (86%) had a symptomatic ICA stenosis. The median post-CEA ICA flow was 170 mL/min [IQR 140–242; range 40–800]. Post-CEA ICA flow was not associated with age, sex, comorbidities, symptomatic stenosis, left ventricular ejection fraction (LVEF), or ipsilateral stenosis severity but was significantly higher in patients requiring intraluminal shunt placement during CEA (*p* = 0.010) as well as in those with contralateral steno-occlusive disease (*p* < 0.001). **Conclusions**: TTF is a practical intraoperative tool for quantitative flow assessment during CEA. Higher post-CEA ICA flow in patients with an intraoperative shunt placement or with contralateral steno-occlusive disease may reflect a compensatory hyperperfusion, as the ipsilateral ICA serves as a key supplier when collateral pathways are incomplete.

## 1. Introduction

Atherosclerotic cerebrovascular steno-occlusive disease accounts for approximately 25% of all ischemic strokes [1]. The prevalence of ICA stenosis increases significantly with age, with rates rising to 6–15% in individuals over 65 years. Carotid endarterectomy (CEA) is a well-established treatment for high-grade symptomatic and asymptomatic ICA stenosis. Large multicenter studies have made secondary and tertiary prevention of carotid stenosis a prime example of evidence-based medicine [2,3,4,5,6]. However, given its preventive nature, extremely low rates of perioperative complications are tolerated. Adverse events, such as ischemic complications, may arise from embolism, intraluminal thrombosis, or hypoperfusion during cross-clamping [7]. Additionally, technical errors such as residual stenosis or residual intraluminal thrombosis may cause postoperative or late complications [8]. Several monitoring techniques, including neuromonitoring, sonography and Transit Time Flowmeter (TTF), are therefore used intraoperatively to ensure the quality of the CEA [9].

TTF is an easy-to-use, non-invasive and safe intraoperative tool that allows for the quantification of blood flow (in mL/min) in vessels, with low artifact rates as well as precise and reproducible measurements. This ensures the quality of the arterial wall reconstruction [10]. TTF is widely used in cardiothoracic surgery, particularly to investigate the graft flow after a cardiac bypass [11,12]. In our institution, in addition to carotid endarterectomies, we use TTFs for cerebral bypasses and intracranial aneurysm surgeries, where the Charbel transit-time flow probe is utilized. Our group has previously reported its experience on the use of TTFs in the measurements of 100 Extracranial–Intracranial flow-augmentation Superficial Temporal Artery to Middle Cerebral Artery Bypasses [13].

For carotid surgery, few studies investigated the use of TTFs so far. These were mostly in the context of quality control. Cyrek et al. described ICA blood flow measurements with TTF as a quality control to evaluate the technical results of the CEA [14]. Knappich et al. [9] as well as Rockman et al. [15] both described the use of TTF after CEA as an intraoperative completion study. Matsumura et al. further described the use of intraoperative flowmetry to predict cerebral hyperperfusion syndrome [16], while Aleksic et al. [17] primarily focused on extracranial blood flow distribution following CEA.

This study aimed to (1) describe post-CEA internal carotid artery (ICA) flow quantified using transit-time flowmetry (TTF), (2) identify predictors of post-CEA ICA flow, and (3) examine its association with contralateral steno-occlusive disease. We hereby present one of the largest cohorts of CEA procedures with systematically recorded intraoperative post-CEA ICA blood flow measurements using TTF, and the first to specifically focus on potential predictors and the association with contralateral steno-occlusive disease.

## 2. Materials and Methods

### 2.1. Patients and Study Design

This study was conducted in accordance with the Declaration of Helsinki, approved by the Cantonal Ethics Committee of Zurich (Kantonale Ethikkommission Zürich, identifier PB-2017-00093, approval date: 23 February 2017), and is registered at https://clinicaltrials.gov (NCT01628406) [18].

Patients who underwent CEA between January 2019 and July 2024 and had available intraoperative ICA flow measurements obtained with TTF after completion of the endarterectomy and restoration of blood flow were included. ICA stenosis was defined as symptomatic if an ipsilateral ischemic event such as ischemic stroke, retinal artery occlusion, or transient ischemic attack occurred within six months prior to surgery. The ICA stenosis was evaluated preoperatively using duplex ultrasonography (DUS) and computed tomography (CT) with CT angiography (CTA) and/or magnetic resonance imaging (MRI) with MR angiography (MRA). The degree of ICA stenosis was classified according to the North American Symptomatic Carotid Endarterectomy Trial (NASCET) criteria [19].

Demographic and clinical data were extracted from electronic medical records at hospital admission and during the inpatient stay. The National Institutes of Health Stroke Scale (NIHSS) and modified Rankin Scale (mRS) scores at admission and before surgery were reviewed to assess functional status and outcome. In addition to common cerebrovascular risk factors and comorbidities (current smoking, arterial hypertension, dyslipidemia, diabetes mellitus, coronary artery disease, and peripheral artery disease), additional parameters potentially influencing ICA flow were collected, including atrial fibrillation, left ventricular ejection fraction (LVEF), and the degree of contralateral ICA stenosis according to NASCET criteria. Post-CEA ICA blood flow in mL/min obtained by TTF was extracted from the operative reports. Furthermore, information on the CEA surgical technique, including the closure method (primary closure, patch, or eversion technique) and the use of an intraluminal shunt in cases of a drop in neuromonitoring potentials, was also obtained from the operative reports.

### 2.2. Surgical Technique and Perioperative Protocol

All patients received either 100 mg Aspirin, 75 mg Clopidogrel, or dual antiplatelet therapy prior to surgery. All surgeries were conducted under general anesthesia and under continuous electrophysiological monitoring of motor and sensory evoked potentials (MEP and SEP) to detect intraoperative ischemic complications. All procedures were performed by the same two senior surgeons (G.E. and L.R.) under the microscope.

Multiple electrode aggregometry (Multiplate^®^ analyzer, Roche Diagnostics, Mannheim, Germany) was performed pre- or intraoperatively to confirm patient responsiveness to the administered antiplatelet therapy (aspirin and/or clopidogrel).

Following cervical dissection, the ICA, common carotid artery (CCA), and external carotid artery (ECA) were clamped. Systolic blood pressure was maintained at 160–180 mmHg during cross-clamping and reduced to 100–130 mmHg upon clamp removal.

Intraluminal shunts were placed when a significant drop in somatosensory or motor evoked potential (SEP/MEP) was detected during cross-clamping. Following endarterectomy, the arteriotomy closure was performed using a primary suture, a patch-technique, or the eversion-technique. The choice of the technique depended on the angioanatomy.

### 2.3. Intraoperative Quality Assessment Following Endarterectomy

**Figure 1:**
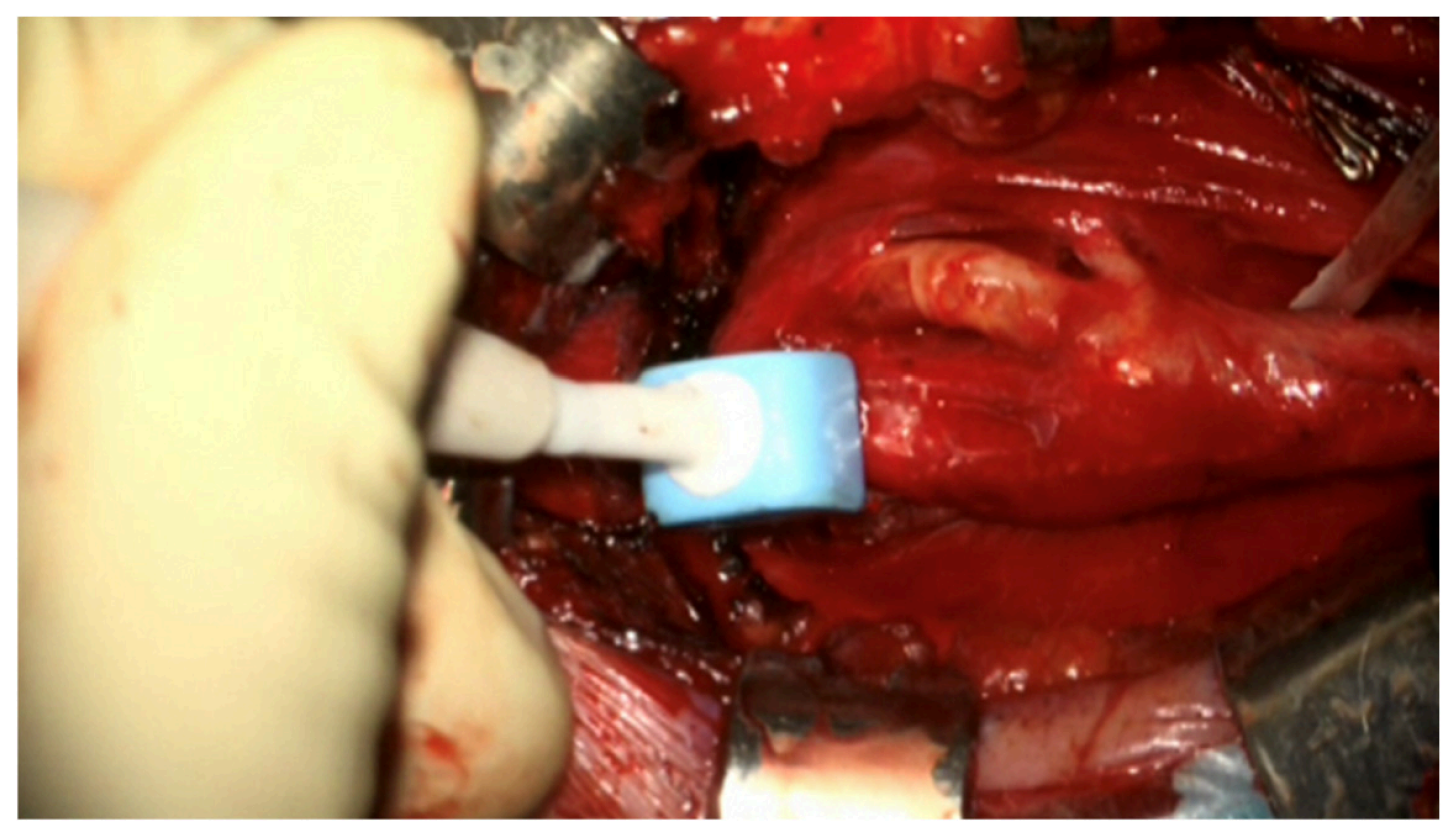
Illustration of intraoperative ICA blood flow measurement with the 6 mm transonic probe (Transonic Systems Inc., 34 Dutch Mill Road, Ithaca, NY 14850, USA) after a carotid endarterectomy.

A duplex sonography was performed intra-operatively as quality control to assess the patency of the ICA, CCA and ECA, and to exclude the presence of intimal flaps, intraluminal thrombosis or stenosis. Afterwards, we used TTF to measure the flow in the ICA as quantitative control, as illustrated in Figure 1.

### 2.4. Intraoperative Flow Measurements with the Transit Time Flowmeter

The TTF probe is matched to the appropriate size of the vessel diameter and is placed perpendicularly to the vessel. The tip is irrigated with Ringer’s solution while the probe is in gentle contact with the vessel probe at all three contact points. In our institution, we use the 6 mm transonic flowmeter (Transonic Systems Inc., 34 Dutch Mill Road, Ithaca, NY 14850, USA). There are different sizes of flow probes: for the carotid artery, a 6 mm flow probe is routinely used, and a 3 mm probe only rarely. Postoperatively, patients were monitored in the intermediate or intensive care unit until postoperative day 1 and then transferred to the ward. Aspirin therapy was continued, while clopidogrel was discontinued in patients previously receiving dual antiplatelet therapy. If multiple electrode aggregometry (Multiplate^®^ analyzer) indicated low responsiveness to aspirin, the aspirin dose was increased. In cases of aspirin non-responsiveness, aspirin was discontinued and clopidogrel was initiated instead.

### 2.5. Assessment of the Collateral Circulation

In a subanalysis, collateral circulation was evaluated in patients who showed a decrease in SEP/MEP requiring intraoperative intraluminal shunt placement.

Collateral pathways were evaluated using transcranial color-coded Doppler sonography (TCD) as described previously [13,20,21,22]: primary collaterals included the anterior communicating artery (ACOM; reversed flow in ipsilateral A1) and posterior communicating artery (PCOM; >50% velocity increase in ipsilateral P1). Secondary collaterals included the ophthalmic artery (OA; reversed periorbital flow) and leptomeningeal (LPM) collaterals (>30% peak systolic velocity increase in ipsilateral P2 or absolute peak systolic velocity >85 cm/s). Furthermore, we assessed whether the Circle of Willis (CoW) of these patients was complete based on the preoperative MRA/CTA.

### 2.6. Statistical Analysis

Numerical variables are presented as mean ± standard deviation or, in the case of non-normal distribution, as median (1st quartile, 3rd quartile). Categorical variables are presented as a number (%). A univariable linear regression analysis was conducted using the mean ICA flow as the dependent variable and the following patient characteristics as independent variable: age, sex, smoking status, hypertension, dyslipidemia, diabetes mellitus, coronary artery disease, peripheral artery occlusive disease, chronic kidney disease, symptomatic stenosis, mRS at admission, atrial fibrillation, left ventricular ejection fraction (LVEF), contralateral steno-occlusive disease and placement of intraluminal shunt. Results of the linear regression are presented as the beta coefficient (95% Confidence Interval [CI]). All statistical analyses were performed with RStudios (version 2025.09.1; Posit Software, PBC, Boston, MA, USA). A *p* value < 0.05 was considered statistically significant. Individual post-CEA ICA flow values for all 196 cases are reported in Supplementary Table S1.

## 3. Results

### 3.1. Demographics

Data from 196 CEAs performed in 190 patients (median age 74 [67–81] years; 25% female) were included. The indication for surgery was always established in an interdisciplinary way. In six patients, CEA was also performed on the contralateral side, with a minimum interval of three months between interventions. Most patients (86%) presented with a symptomatic ischemic event. The median time from symptom onset to CEA in these cases was 9 days (IQR 6–17). Patient baseline characteristics are summarized in Table 1. Most patients (98%) had at least a 50% ICA stenosis according to NASCET criteria as measured in a DUS. Four patients presented with 40% NASCET stenosis and signs of plaque vulnerability. Median NASCET stenosis was 70% (IQR 60–80%). A contralateral ICA stenosis was present in 106 of the cases. Specific signs of plaque vulnerability on carotid ultrasound of the ICA were described in only 16 patients, as listed in Table 1.

### 3.2. Intraoperative ICA Flow Measurement and Its Predictors

TTF measurements were performed quickly and easily, always requiring less than one minute. No vascular avulsion or inadvertent tissue trauma occurred during probe positioning or use. The median post-CEA ICA flow was 170 mL/min [IQR 140–242; range 40–800].

Table 2 summarizes the effect of patient characteristics on post-CEA ICA flow. In the univariable analysis, we found a significant association between male sex and mean ICA flow (*p* = 0.042). However, after adjusting for LVEF, this result was no longer significant, beta 20 (−37, 77), *p* = 0.5. The median LVEF for all patients was 58% (IQR 54, 62). Male patients had a median LVEF of 57% (54, 62), whereas female patients showed a higher median LVEF of 62% (56, 64) (*p* = 0.005), although data were not available in 40% of patients (*n* = 78).

None of the cerebrovascular risk factors (current smoking, arterial hypertension, dyslipidaemia, diabetes mellitus) or vascular comorbidities (coronary artery disease, peripheral artery disease, and chronic kidney disease) had a significant influence on mean post-CEA ICA flow. Furthermore, neither mRS on admission nor symptomatic stenosis was associated with mean ICA flow. In addition, neither LVEF nor atrial fibrillation showed any significant effect on ICA flow after blood flow restoration post-clamping.

However, patients requiring intraoperative intraluminal shunt placement showed a significantly higher mean ICA flow (*p* = 0.010), with a median of 250 mL/min (IQR 210–320) compared with 170 mL/min (IQR 133–230) in those without shunt placement.

**Figure 2:**
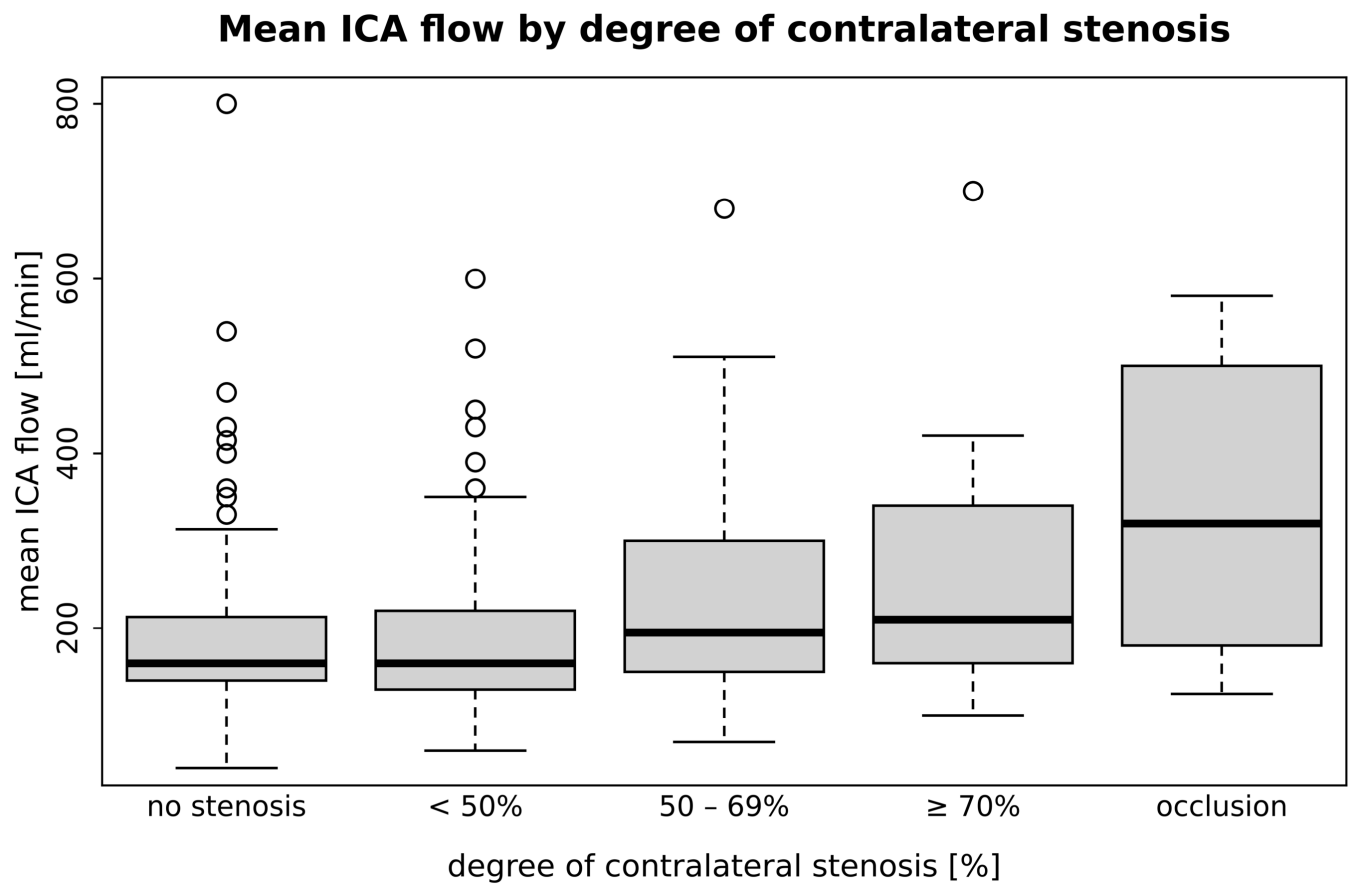
Association between the NASCET degree of contralateral ICA stenosis and ipsilateral post-CEA ICA flow. NASCET = North American Symptomatic Carotid Endarterectomy Trial; ICA = internal carotid artery; CEA = carotid endarterectomy.

There was no association between the degree of NASCET stenosis on the ipsilateral side and ICA flow (β = 5.7; 95% CI, −28 to 39; *p* = 0.70). However, a significantly higher ICA flow was observed with increasing NASCET degree of stenosis on the contralateral side (*p* < 0.001). As illustrated in Figure 2, post-CEA ICA flow increased progressively with the severity of contralateral steno-occlusive disease.

Supplementary Table S1 shows every individual intraoperative post-CEA ICA flow. Flow values are stratified by the severity of contralateral steno-occlusive disease and by the use of an intraoperative intraluminal shunt. Median post-CEA ICA flow was 240 mL/min (IQR 169–375; range 100–700) in the cases with severe contralateral steno-occlusive disease (stenosis ≥ 70% or occlusion), compared with 160 mL/min (IQR 133–230; range 40–800) in the cases with non-severe contralateral disease (no, low-grade, or moderate-grade stenosis). Across the individual NASCET categories, median post-CEA ICA flow rose from 160 mL/min in cases without contralateral stenosis to 194 mL/min in cases with moderate-grade stenosis, 210 mL/min with high-grade stenosis, and 322 mL/min in cases with contralateral ICA occlusion.

### 3.3. Subanalysis of Patients Requiring an Intraluminal Shunt Placement During CEA

In the nine patients requiring intraoperative shunt placement, preoperative findings from DUS including TCD and CTA and/or MRA were evaluated for the presence and activation of primary and secondary collateral pathways, the completeness of the CoW, and the degree of ipsilateral and contralateral steno-occlusive disease. These findings are detailed in Table 3. In this subgroup, preoperative imaging revealed varying combinations of leptomeningeal collateral activation, incomplete Circle of Willis, and/or contralateral high-grade steno-occlusive disease, as detailed below.

Two patients (Patients 2 and 8), both with 70% ipsilateral NASCET stenosis, showed preoperative activation of leptomeningeal collaterals in combination with at least one additional primary or secondary collateral pathway on TCD. One of them presented with a contralateral ICA occlusion, and the other with bilateral vertebral artery occlusion together with a contralateral 80% NASCET stenosis, in both cases resulting in insufficient collateralization during cross-clamping.

Three further patients (Patients 1, 5, and 9) with high-grade ipsilateral ICA stenosis (one with 70% and two with 80% NASCET) showed an activation of both primary and secondary collateral pathways on TCD, but no leptomeningeal collaterals. Two of these patients had a hypoplastic or incomplete CoW affecting the anterior circulation, and one exhibited a hypoplastic PCOM.

In the four patients (Patients 3, 4, 6, and 7) with 50% ipsilateral ICA stenosis according to the NASCET criteria, no activation of the primary or secondary collateral pathways was observed on TCD. Patients 3 and 4 demonstrated an incomplete CoW involving both the anterior and posterior circulation on MRA. Patient 6 had an additional high-grade ECA stenosis as well as a P1 stenosis, and Patient 7 presented with a contralateral ICA occlusion.

However, these observations are based on only nine patients. This subanalysis is therefore descriptive and exploratory. The patterns reported here should be regarded as hypothesis-generating and require confirmation in larger, prospectively collected cohorts.

### 3.4. Perioperative Stroke, Death, Myocardial Infarction and ICA Restenosis

The lowest post-CEA ICA flow measured was 40 mL/min and the highest was 800 mL/min. Neither of these two patients developed a postoperative complication.

An intracerebral hemorrhage occurred postoperatively in a patient with cerebral amyloid angiopathy and hypertension. The post-CEA ICA flow value was 132 mL/min, which is within the range of our cohort. He was initially treated in the intensive care unit. His treatment goal was then changed to palliative care, and he died 19 days postoperatively. A second patient died of a postoperative myocardial infarction with cardiac arrest and pulseless electrical activity, refractory to cardiopulmonary resuscitation (post-CEA ICA flow: 180 mL/min). A third patient died from complications of status epilepticus, most likely attributable to a preexisting ischemic stroke that showed slight postoperative progression (post-CEA ICA flow of 310 mL/min). Postoperative CT perfusion imaging showed no signs of hyperperfusion. No other patient of our cohort sustained an ischemic stroke or intracerebral haemorrhage within three months of discharge. Death and stroke rate was 1.6% in our consecutive series of which 86% of patients had CEA for symptomatic ICA stenosis.

Furthermore, two patients underwent revision surgery for asymptomatic ICA restenosis. In the first, postoperative CT angiography demonstrated a 70% restenosis. ICA flow was 360 mL/min at the end of the initial procedure and 220 mL/min after revision. In the second, the restenosis was caused by ICA kinking. Flow was 180 mL/min at the end of the initial procedure and remained unchanged after revision. Postoperative imaging confirmed resolution of the stenosis in both. Neither patient developed an ischemic stroke, transient ischemic attack, or new neurological deficit postoperatively or during follow-up, and both had a favorable outcome (mRS 0).

As the registry was prospective with consecutive patient enrolment, adverse events were identified and documented at the time of occurrence rather than through retrospective chart review.

Perioperative events in this cohort were not preceded by a consistent pattern of abnormal intraoperative TTF values. However, given the low number of events, these findings remain descriptive.

## 4. Discussion

In this study, we present one of the largest single-center cohorts of 196 consecutive cases with systematically recorded intraoperative ICA blood flow measurements using TTF after CEA. Post-CEA ICA flow showed a wide flow range (median 170 mL/min; [IQR 140–242; range 40–800]). These findings are in line with previously reported post-CEA ICA flow values ranging from 165 to 300 mL/min, measured using either intraoperative TTF, intraoperative duplex scanning or postoperative MR-Angiography [16,23,24]. Indeed, Matsumura et al. also described a median ICA flow of 170 mL/min (IQR 158.5–257). Aleksic et al. obtained a median ICA flow of 240 mL/min (IQR 187–309), whereas Cyrek et al. described a mean ICA flow of 216 mL/min (range, 90–550). The wide range of post-CEA ICA flow values in our cohort likely reflects individual differences in vessel size and collateral circulation.

In our study, none of the demographic characteristics, cerebrovascular risk factors or vascular comorbidities showed a significant association with mean post-CEA ICA flow or served as independent predictors. Male sex initially appeared to influence post-CEA ICA flow; however, this association was not confirmed after adjustment for LVEF, suggesting that differences in cardiac output may partly explain the observed variability in ICA flow between men and women. Interpretation of this finding is limited by missing LVEF data in 40% of patients, which may have affected the analysis. Recent studies [25,26] have shown that higher LVEF correlates positively with increased ICA flow, reflecting the influence of cardiac output on cerebral perfusion. In contrast, compensatory cerebral autoregulation mechanisms help maintain adequate cerebral blood flow in cases of acute LVEF decline. Of note, in our study, female patients had a higher median LVEF (62% vs. 57%, *p* = 0.005), consistent with known sex-related differences showing higher baseline cardiac function [27,28]. Moreover, recent studies [29] have shown that women have higher baseline cerebral blood flow despite smaller vessel diameters; however, with increasing age, ICA flow velocities decline more markedly in women, while the reduction is less pronounced in men.

There was no association between the degree of ipsilateral NASCET stenosis and post-CEA ICA flow, contrasting with the findings of Dörfler et al., who reported an influence of preoperative ipsilateral ICA stenosis on post-CEA perfusion in a cohort of 26 patients [30]. These discrepancies may be explained by methodological differences, as Dörfler et al. used perfusion-weighted MRI and assessed flow three months after CEA rather than intraoperatively. The observed significantly higher ICA flow with increasing contralateral NASCET stenosis (*p* < 0.001) is most likely explained by compensatory hyperperfusion secondary to contralateral steno-occlusive disease. This effect is well recognized in duplex ultrasonography, where moderate (NASCET 50–69%) and particularly severe (NASCET 70–99%) contralateral stenosis can lead to artificially elevated peak systolic velocities on the ipsilateral side due to compensatory flow increase [31].

In the nine patients requiring intraoperative shunt placement, preoperative imaging revealed limited collateral capacity associated with an incomplete CoW and/or contralateral high-grade steno-occlusive disease. The significantly higher post-CEA ICA flow observed in these patients (*p* = 0.010) may reflect compensatory hyperperfusion, as the ipsilateral ICA serves as the dominant supplier when collateral pathways are incomplete. During cross-clamping, such insufficient collaterals may therefore result in a relevant drop in intraoperative neuromonitoring signals (SEP and/or MEP). Given that only nine patients required a shunt, this interpretation remains hypothesis-generating.

Cerebral hyperperfusion syndrome (CHS) may occur after carotid revascularization and may result in brain oedema or intracerebral hemorrhage if not treated properly. It is attributed to the abrupt restoration of perfusion pressure in a chronically hypoperfused hemisphere in which autoregulatory reserve is exhausted [32]. Matsumura et al. used transit time flowmetry to investigate this phenomenon and found that the post-CEA ICA flow alone did not discriminate between patients with and without cerebral hyperperfusion, whereas the absolute intraoperative flow increase (post- minus pre-CEA flow) did, with a cut-off of 81 mL/min [16]. In our cohort, pre-CEA flow of the ICA was deliberately not measured to avoid manipulation of the diseased vessel before clamping, so this increase could not be calculated. Consistent with Matsumura’s observation, the patient with the highest flow (800 mL/min) had an uneventful course, whereas the patient with a postoperative intracerebral hemorrhage had a flow value of 132 mL/min, which is within the range of the overall cohort.

Some limitations to this study should be acknowledged. First, this was a single-center analysis, which inherently limits causal interpretation and generalizability to other surgical settings. Nevertheless, intraoperative blood pressure values were maintained within standardized ranges—typically <140 mmHg before cross-clamping, 160–180 mmHg during cross-clamping, and 100–130 mmHg after declamping—to minimize ICA flow variability. Second, although it would have been valuable to include pre-CEA ICA flow measurements to assess the degree of flow change, this was intentionally avoided to prevent unnecessary manipulation of the ICA before clamping and reduce embolic risk. Third, LVEF data were missing in approximately 40% of patients, which may have influenced the analysis of cardiac–cerebral flow relationships. Lastly, the inclusion of six patients who underwent bilateral CEA may have introduced a minor intra-patient dependency bias. Despite these limitations, this study provides valuable real-world data, systematically evaluating intraoperative ICA flow during CEA and its clinical relevance.

## 5. Conclusions

This study reports on one of the largest cohorts of CEA procedures where ICA blood flow post-endarterectomy was measured intraoperatively using TTF. It is the first to specifically focus on potential predictors and the association with contralateral steno-occlusive disease. The findings highlight the value of TTF as a practical intraoperative tool for quantitative assessment of ICA flow during CEA, thus providing both objective quality control and insight into cerebral perfusion. Higher post-CEA ICA flow in patients with an intraoperative shunt placement or with contralateral steno-occlusive disease may reflect compensatory hyperperfusion, as the ipsilateral ICA serves as a key supplier when collateral pathways are incomplete.

## Figures and Tables

**Table 1:** Baseline characteristics of the study population.

Baseline Characteristic	Overall Patients *n* = 190 *
age	74 (67, 81)
Female	47 (25%)
Vascular risk factors	
Current smoking	54 (28%)
Hypertension	140 (74%)
Dyslipidemia	103 (54%)
Diabetes mellitus	52 (27%)
Comorbidities	
Coronary artery disease	50 (26%)
Peripheral artery occlusive disease	29 (15%)
Chronic kidney disease	29 (15%)
Atrial fibrillation	23 (12%)
LVEF %	58 (54, 62)
Missing/LVEF n.a.	78 (40%)
Type of presentation	
Asymptomatic ICA stenosis	27 (14%)
Symptomatic ICA stenosis	163 (86%)
Retinal artery occlusion	4 (2%)
TIA	36 (19%)
Ischemic stroke	123 (65%)
TOAST	
1	187 (98%)
5	3 (1.5%)
NASCET ipsilateral ICA stenosis	
<50%	4 (2%)
50–69%	69 (36%)
≥70%	117 (62%)
mRS on admission	
0	84 (45%)
1	47 (25%)
2	30 (13%)
3	14 (8%)
4	12 (7%)
5	3 (2%)
mRS preoperative	
0	91 (48%)
1	59 (31%)
2	27 (14%)
3	13 (7%)
4	0 (0%)
5	0 (0%)
NIHSS preoperative	
0	110 (58%)
1–5	72 (38%)
6–14	8 (4%)
NASCET of contralateral_ICA stenosis	
No stenosis	84 (44%)
<50%	61 (32%)
50–69%	20 (11%)
70–99%	15 (8%)
Occlusion	10 (4%)
Plaque_vulnerability in DUS	
floating thrombus	16 (8.5%)
soft plaque	5 (2.5%)
thrombus	3 (1.5%)
ulceration	4 (2%)

* Data are presented as median (IQR) or *n* (%), as appropriate. ICA = internal carotid artery; CEA = carotid endarterectomy; DUS = duplex ultrasound; LVEF = left ventricular ejection fraction; TIA = transient ischemic attack; TOAST = Trial of Org 10172 in Acute Stroke Treatment; NASCET = North American Symptomatic Carotid Endarterectomy Trial; mRS = modified Rankin Scale; NIHSS = National Institutes of Health Stroke Scale; CT = computed tomography.

**Table 2:** Univariable linear regression assessing the association between clinical variables and mean ICA flow *.

Variable	β-Coefficient (95% CI)	*p*-Value
Age	−1.47 (−3.41, 0.48)	0.15
Male sex	20 (−37, 77)	0.5
Current Smoking	14.19 (−26.85, 55.22)	0.50
Hypertension	20.12 (−20.15, 60.38)	0.33
Dyslipidemia	14.27 (−21.21, 49.75)	0.43
Diabetes mellitus	−8.55 (−48.18, 31.07)	0.67
Coronary artery disease	−9.86 (−50.20, 30.49)	0.63
Peripheral artery occlusive disease	17.97 (−31.85, 67.79)	0.48
Chronic kidney disease	1.69 (−12.02, 15.39)	0.81
Symptomatic stenosis	26.5 (−25.3, 78.3)	0.31
mRS at admission	−4.9 (−18.6, 8.8)	0.48
Atrial fibrillation	31.39 (−15.73, 78.51)	0.19
LVEF	−0.11 (−3.38, 3.16)	0.95
Contralateral steno-occlusive disease	28 (13, 43)	**<0.001**
**Placement of intraluminal shunt**	110 (27, 193)	**0.010**

* β = beta coefficient; CI = confidence interval; ICA = internal carotid artery; LVEF = left ventricular ejection fraction; mRS = modified Rankin Scale.

**Table 3:** Preoperative TCD, MRA, and carotid DUS findings: collateral flow, circle of Willis completeness, and ipsilateral and contralateral stenosis *.

Pat.	Ipsilateral ICA Stenosis NASCET (%)	ACOM Collateral	PCOM Collateral	OA Collateral	LPM Collateral	Incomplete Circle of Willis (CTA/MRA)	Contralateral ICA Stenosis NASCET (%)	Ipsilateral ECA Stenosis High-Grade
1	80%	No	Yes	Yes	No	Hypoplastic ipsilat. A1	No	No
2	70%	No	Yes	No	Yes	No	Occlusion	Yes
3	50%	No	No	No	No	Absence of ACOM (variant), no PCOM	<50%	No
4	50%	No	No	No	No	Hypoplastic ACOM, no ipsilat. PCOM	<50%	No
5	80%	No	Yes	Yes	No	Hypoplastic contralat. A1	No	No
6	50%	No	No	No	No	Absence of contralateral A1, high-grade bilateral P1 stenosis	<50%	Yes
7	50%	No	No	No	No	No	Occlusion	No
8	70%	No	No	Yes	Yes	Bilateral VA occlusion	80%	No
9	70%	No	Yes	Yes	No	Hypoplastic ipsilat. PCOM	No	No

* ICA, internal carotid artery; ECA, external carotid artery; NASCET, North American Symptomatic Carotid Endarterectomy Trial; ACOM, anterior communicating artery; PCOM, posterior communicating artery; OA, ophthalmic artery; LPM, leptomeningeal; VA, vertebral artery.

## Data Availability

The underlying data are available upon reasonable request to the corresponding author. The data is not publicly available due to data protection and privacy.

## Supplementary Materials

The following supporting information can be downloaded at: https://www.mdpi.com/article/10.3390/brainsci16101136/s1, Table S1: Intraoperative post-CEA ICA flow (mL/min) measured by intraoperative flowmetry, listed for every case, categorized by severity of contralateral stenoocclusive disease (severe = high-grade (≥70%) or occlusion; non-severe = none, low-grade (<50%), or moderate-grade (50–69%) and by use of an intraoperative shunt. In patients who underwent bilateral CEA, the contralateral status refers to the degree of stenosis before the first surgery.

## Abbreviations

The following abbreviations are used in this manuscript:

ACA	Anterior cerebral artery
ACOM	Anterior communicating Artery
CAD	Coronary Artery Disease
CCA	Common Carotid Artery
CEA	Carotid Endarterectomy
CHS	Cerebral Hyperperfusion Syndrome
CI	Confidence Interval
CoW	Circle of Willis
CT	Computed Tomography
CTA	CT Angiography
DUS	Duplex Ultrasonography
ECA	External Carotid Artery
ECASS	European Cooperative Acute Stroke Study
ICA	Internal Carotid Artery
ICH	Intracerebral Hemorrhage
IQR	Interquartile Range
LPM	Leptomeningeal
LVEF	Left Ventricular Ejection Fraction
MCA	Middle Cerebral Artery
MEP	Motor Evoked Potentials
MRA	Magnetic Resonance Angiography
MRI	Magnetic Resonance Imaging
mRS	Modified Rankin Scale
NASCET	North American Symptomatic Carotid Endarterectomy Trial
NIHSS	National Institutes of Health Stroke Scale
OA	Ophthalmic Artery
PAD	Peripheral Artery Disease
HI	Hemorrhagic Infarction
PCOM	Posterior Communicating Artery
PCA	Posterior Cerebral Artery
PH	Parenchymal Hematoma
SD	Standard Deviation
SEP	Somatosensory Evoked Potentials
TCD	Transcranial Doppler Sonography
TIA	Transient Ischemic Attack
TTF	Transit Time Flowmeter
VA	Vertebral Artery
