# Supplementary material for: Intraoperative Measurement of Internal Carotid Artery Blood Flow Using Transit-Time Flowmetry After Carotid Endarterectomy: Results from 196 Consecutive Cases
Source: Brain Sci. 2026 Oct 21;16(10):1136. doi: 10.3390/brainsci16101136 (PMC13702187; doi:10.3390/brainsci16101136)
Supplement: Supplementary file 1 [file brainsci-16-01136-s001.zip › brainsci-4419687-supplementary.pdf]

Supplementary Material

Supplementary Table S1. Intraoperative post-CEA ICA flow (mL/min) measured by intraoperative flowmetry, listed for every case, categorized by severity of contralateral stenoocclusive disease (severe = high-grade (≥ 70%) or occlusion; non-severe = none, low-grade (<50%), or moderate-grade (50–69%) and by use of an intraoperative shunt. In patients who underwent bilateral CEA, the contralateral status refers to the degree of stenosis before the first surgery.

Case	Patient ID	Degree of contralateral steno-occlusive disease	Severity category	Shunt placement	ICA flow (mL/min)
1	49	Occlusion	Severe	Yes	320
2	42	Occlusion	Severe	No	580
3	63	Occlusion	Severe	No	530
4	23	Occlusion	Severe	No	500
5	56	Occlusion	Severe	No	450
6	154	Occlusion	Severe	No	324
7	25	Occlusion	Severe	No	320
8	142	Occlusion	Severe	No	180
9	212	Occlusion	Severe	No	158
10	169	Occlusion	Severe	No	126
11	108	High-grade (≥70%)	Severe	Yes	420
12	118	High-grade (≥70%)	Severe	Yes	170
13	27	High-grade (≥70%)	Severe	No	700
14	20	High-grade (≥70%)	Severe	No	380
15	105	High-grade (≥70%)	Severe	No	360
16	121	High-grade (≥70%)	Severe	No	330
17	158	High-grade (≥70%)	Severe	No	260
18	46	High-grade (≥70%)	Severe	No	220
19	146	High-grade (≥70%)	Severe	No	210
20	158	High-grade (≥70%)	Severe	No	210
21	15	High-grade (≥70%)	Severe	No	180
22	155	High-grade (≥70%)	Severe	No	168
23	172	High-grade (≥70%)	Severe	No	150
24	15	High-grade (≥70%)	Severe	No	130
25	82	High-grade (≥70%)	Severe	No	130
26	135	High-grade (≥70%)	Severe	No	130
27	74	High-grade (≥70%)	Severe	No	100
28	30	Moderate-grade (50–69%)	Non-severe	No	680
29	32	Moderate-grade (50–69%)	Non-severe	No	510
30	141	Moderate-grade (50–69%)	Non-severe	No	500
31	53	Moderate-grade (50–69%)	Non-severe	No	330
32	165	Moderate-grade (50–69%)	Non-severe	No	330
33	110	Moderate-grade (50–69%)	Non-severe	No	300
34	139	Moderate-grade (50–69%)	Non-severe	No	250
35	137	Moderate-grade (50–69%)	Non-severe	No	230
36	199	Moderate-grade (50–69%)	Non-severe	No	210
37	97	Moderate-grade (50–69%)	Non-severe	No	200
38	157	Moderate-grade (50–69%)	Non-severe	No	194
39	151	Moderate-grade (50–69%)	Non-severe	No	170
40	126	Moderate-grade (50–69%)	Non-severe	No	160

Case	Patient ID	Degree of contralateral steno-occlusive disease	Severity category	Shunt placement	ICA flow (mL/min)
41	213	Moderate-grade (50–69%)	Non-severe	No	160
42	20	Moderate-grade (50–69%)	Non-severe	No	150
43	34	Moderate-grade (50–69%)	Non-severe	No	150
44	166	Moderate-grade (50–69%)	Non-severe	No	140
45	61	Moderate-grade (50–69%)	Non-severe	No	130
46	105	Moderate-grade (50–69%)	Non-severe	No	130
47	131	Moderate-grade (50–69%)	Non-severe	No	100
48	145	Moderate-grade (50–69%)	Non-severe	No	100
49	152	Moderate-grade (50–69%)	Non-severe	No	70
50	54	Low-grade (<50%)	Non-severe	Yes	310
51	109	Low-grade (<50%)	Non-severe	Yes	210
52	117	Low-grade (<50%)	Non-severe	Yes	160
53	96	Low-grade (<50%)	Non-severe	No	600
54	175	Low-grade (<50%)	Non-severe	No	520
55	1	Low-grade (<50%)	Non-severe	No	450
56	17	Low-grade (<50%)	Non-severe	No	430
57	174	Low-grade (<50%)	Non-severe	No	390
58	84	Low-grade (<50%)	Non-severe	No	360
59	211	Low-grade (<50%)	Non-severe	No	350
60	176	Low-grade (<50%)	Non-severe	No	300
61	14	Low-grade (<50%)	Non-severe	No	250
62	50	Low-grade (<50%)	Non-severe	No	250
63	138	Low-grade (<50%)	Non-severe	No	250
64	163	Low-grade (<50%)	Non-severe	No	240
65	86	Low-grade (<50%)	Non-severe	No	230
66	107	Low-grade (<50%)	Non-severe	No	230
67	123	Low-grade (<50%)	Non-severe	No	230
68	119	Low-grade (<50%)	Non-severe	No	220
69	47	Low-grade (<50%)	Non-severe	No	210
70	11	Low-grade (<50%)	Non-severe	No	200
71	59	Low-grade (<50%)	Non-severe	No	200
72	64	Low-grade (<50%)	Non-severe	No	190
73	82	Low-grade (<50%)	Non-severe	No	190
74	4	Low-grade (<50%)	Non-severe	No	180
75	114	Low-grade (<50%)	Non-severe	No	180
76	200	Low-grade (<50%)	Non-severe	No	180
77	159	Low-grade (<50%)	Non-severe	No	172
78	100	Low-grade (<50%)	Non-severe	No	170
79	133	Low-grade (<50%)	Non-severe	No	170
80	170	Low-grade (<50%)	Non-severe	No	170
81	85	Low-grade (<50%)	Non-severe	No	160
82	91	Low-grade (<50%)	Non-severe	No	160
83	10	Low-grade (<50%)	Non-severe	No	150
84	71	Low-grade (<50%)	Non-severe	No	150
85	95	Low-grade (<50%)	Non-severe	No	150
86	127	Low-grade (<50%)	Non-severe	No	150
87	134	Low-grade (<50%)	Non-severe	No	150
88	208	Low-grade (<50%)	Non-severe	No	148

Case	Patient ID	Degree of contralateral steno-occlusive disease	Severity category	Shunt placement	ICA flow (mL/min)
89	160	Low-grade (<50%)	Non-severe	No	145
90	198	Low-grade (<50%)	Non-severe	No	144
91	67	Low-grade (<50%)	Non-severe	No	140
92	103	Low-grade (<50%)	Non-severe	No	140
93	104	Low-grade (<50%)	Non-severe	No	140
94	161	Low-grade (<50%)	Non-severe	No	132
95	106	Low-grade (<50%)	Non-severe	No	130
96	153	Low-grade (<50%)	Non-severe	No	130
97	164	Low-grade (<50%)	Non-severe	No	130
98	173	Low-grade (<50%)	Non-severe	No	125
99	8	Low-grade (<50%)	Non-severe	No	120
100	70	Low-grade (<50%)	Non-severe	No	120
101	73	Low-grade (<50%)	Non-severe	No	120
102	94	Low-grade (<50%)	Non-severe	No	120
103	167	Low-grade (<50%)	Non-severe	No	114
104	148	Low-grade (<50%)	Non-severe	No	110
105	204	Low-grade (<50%)	Non-severe	No	105
106	89	Low-grade (<50%)	Non-severe	No	100
107	99	Low-grade (<50%)	Non-severe	No	100
108	130	Low-grade (<50%)	Non-severe	No	100
109	144	Low-grade (<50%)	Non-severe	No	100
110	216	Low-grade (<50%)	Non-severe	No	93
111	112	Low-grade (<50%)	Non-severe	No	80
112	52	Low-grade (<50%)	Non-severe	No	60
113	39	No stenosis	Non-severe	Yes	800
114	16	No stenosis	Non-severe	Yes	250
115	58	No stenosis	Non-severe	Yes	230
116	51	No stenosis	Non-severe	No	540
117	57	No stenosis	Non-severe	No	470
118	80	No stenosis	Non-severe	No	430
119	31	No stenosis	Non-severe	No	410
120	19	No stenosis	Non-severe	No	400
121	43	No stenosis	Non-severe	No	360
122	69	No stenosis	Non-severe	No	350
123	75	No stenosis	Non-severe	No	330
124	55	No stenosis	Non-severe	No	310
125	9	No stenosis	Non-severe	No	300
126	44	No stenosis	Non-severe	No	300
127	62	No stenosis	Non-severe	No	300
128	210	No stenosis	Non-severe	No	270
129	48	No stenosis	Non-severe	No	250
130	60	No stenosis	Non-severe	No	250
131	22	No stenosis	Non-severe	No	230
132	87	No stenosis	Non-severe	No	230
133	2	No stenosis	Non-severe	No	220
134	124	No stenosis	Non-severe	No	210
135	13	No stenosis	Non-severe	No	200
136	38	No stenosis	Non-severe	No	200

Case	Patient ID	Degree of contralateral steno-occlusive disease	Severity category	Shunt placement	ICA flow (mL/min)
137	88	No stenosis	Non-severe	No	200
138	171	No stenosis	Non-severe	No	200
139	5	No stenosis	Non-severe	No	190
140	65	No stenosis	Non-severe	No	190
141	26	No stenosis	Non-severe	No	180
142	37	No stenosis	Non-severe	No	180
143	40	No stenosis	Non-severe	No	180
144	41	No stenosis	Non-severe	No	180
145	45	No stenosis	Non-severe	No	180
146	72	No stenosis	Non-severe	No	180
147	143	No stenosis	Non-severe	No	180
148	156	No stenosis	Non-severe	No	180
149	209	No stenosis	Non-severe	No	180
150	12	No stenosis	Non-severe	No	170
151	113	No stenosis	Non-severe	No	170
152	140	No stenosis	Non-severe	No	170
153	215	No stenosis	Non-severe	No	170
154	149	No stenosis	Non-severe	No	168
155	7	No stenosis	Non-severe	No	160
156	68	No stenosis	Non-severe	No	160
157	79	No stenosis	Non-severe	No	160
158	86	No stenosis	Non-severe	No	160
159	147	No stenosis	Non-severe	No	160
160	214	No stenosis	Non-severe	No	160
161	177	No stenosis	Non-severe	No	159
162	3	No stenosis	Non-severe	No	150
163	6	No stenosis	Non-severe	No	150
164	18	No stenosis	Non-severe	No	150
165	24	No stenosis	Non-severe	No	150
166	28	No stenosis	Non-severe	No	150
167	33	No stenosis	Non-severe	No	150
168	35	No stenosis	Non-severe	No	150
169	101	No stenosis	Non-severe	No	150
170	120	No stenosis	Non-severe	No	150
171	129	No stenosis	Non-severe	No	150
172	132	No stenosis	Non-severe	No	150
173	201	No stenosis	Non-severe	No	150
174	202	No stenosis	Non-severe	No	150
175	205	No stenosis	Non-severe	No	150
176	122	No stenosis	Non-severe	No	140
177	125	No stenosis	Non-severe	No	140
178	116	No stenosis	Non-severe	No	134
179	36	No stenosis	Non-severe	No	130
180	76	No stenosis	Non-severe	No	130
181	77	No stenosis	Non-severe	No	130
182	78	No stenosis	Non-severe	No	130
183	98	No stenosis	Non-severe	No	130
184	136	No stenosis	Non-severe	No	130

Case	Patient ID	Degree of contralateral steno-occlusive disease	Severity category	Shunt placement	ICA flow (mL/min)
185	207	No stenosis	Non-severe	No	130
186	162	No stenosis	Non-severe	No	128
187	92	No stenosis	Non-severe	No	120
188	102	No stenosis	Non-severe	No	120
189	111	No stenosis	Non-severe	No	120
190	115	No stenosis	Non-severe	No	120
191	150	No stenosis	Non-severe	No	120
192	206	No stenosis	Non-severe	No	120
193	66	No stenosis	Non-severe	No	110
194	83	No stenosis	Non-severe	No	80
195	93	No stenosis	Non-severe	No	70
196	29	No stenosis	Non-severe	No	40

CEA, carotid endarterectomy; ICA, internal carotid artery. Patient ID is shared by both procedures of the six patients who underwent bilateral CEA.
